# Diatom-Specific Oligosaccharide and Polysaccharide Structures Help to Unravel Biosynthetic Capabilities in Diatoms

**DOI:** 10.3390/md13095993

**Published:** 2015-09-18

**Authors:** Bruno Gügi, Tinaïg Le Costaouec, Carole Burel, Patrice Lerouge, William Helbert, Muriel Bardor

**Affiliations:** 1 Laboratoire Glyco-MEV EA 4358, Université de Rouen, Normandie Université, Institut de Recherche et d’Innovation Biomédicale (IRIB), Végétale Agronomie Sol Innovation (VASI), Normandie Université, Faculté des Sciences et Techniques, 76821 Mont-Saint-Aignan, France; E-Mails: bruno.gugi@univ-rouen.fr (B.G.); carole.burel@univ-rouen.fr (C.B.); patrice.lerouge@univ-rouen.fr (P.L.); 2 CNRS, Centre de Recherches sur les Macromolécules Végétales (CERMAV), Université Grenoble Alpes, CERMAV, F-38000 Grenoble, France; E-Mail: tianïg.lecostaouec@cermav.cnrs.fr; 3 Institut Universitaire de France (IUF), 75005 Paris, France

**Keywords:** microalgae, glycan, polysaccharide, diatom, exopolysaccharides, EPS, nucleotide sugars

## Abstract

Diatoms are marine organisms that represent one of the most important sources of biomass in the ocean, accounting for about 40% of marine primary production, and in the biosphere, contributing up to 20% of global CO_2_ fixation. There has been a recent surge in developing the use of diatoms as a source of bioactive compounds in the food and cosmetic industries. In addition, the potential of diatoms such as *Phaeodactylum tricornutum* as cell factories for the production of biopharmaceuticals is currently under evaluation. These biotechnological applications require a comprehensive understanding of the sugar biosynthesis pathways that operate in diatoms. Here, we review diatom glycan and polysaccharide structures, thus revealing their sugar biosynthesis capabilities.

## 1. Introduction

**Figure 1:**
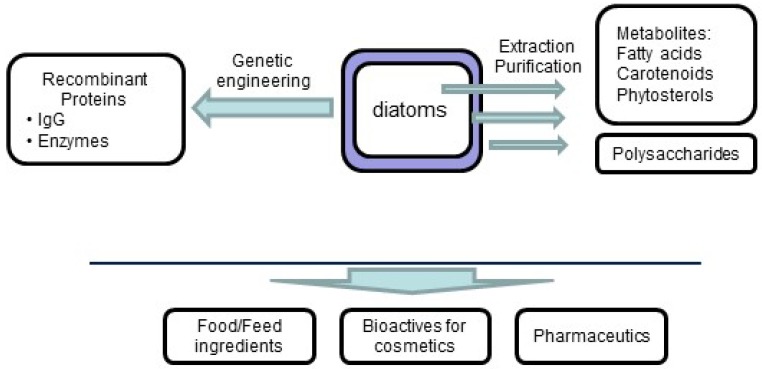
Applications of diatom active compounds in human health and food supplements.

Among ocean phytoplankton, diatoms are highly diverse with an estimated 10^5^ to 10^7^ species [1]. Marine diatoms make up an important group: they contribute to approximately 40% of primary productivity in marine ecosystems and 20% of global carbon fixation [2,3,4,5]. Diatoms also participate in the ocean silica cycle [6,7,8,9], iron cycle [8,10,11,12] and nitrogen cycle [13,14,15,16]. Due to their high diversity and very specific metabolism, diatoms have been used as bio-indicators and filters for controlling and purifying contaminated water [17,18,19,20]. For example, some diatoms such as *Cylindrotheca fusiformis*, *Cyclotella cryptica*, *Phaeodactylum tricornutum*, *Skeletonema costatum,* and *Thalassiosira pseudonana* have been used to absorb high quantities of heavy metals [21,22,23]. Diatoms are also used in nanotechnology to produce living nano-scale structures because they can build a silica shell at room temperature from a very small amount of silica dissolved in water [24,25,26,27]. In parallel, diatoms have been explored as sources of bioactive metabolites. Such compounds have many uses in the food industry. For example, diatoms have long been used as feedstock in aquaculture [28] and more recently in human health and food supplements (Figure 1).

Given their ability to produce carotenoids, phytosterols, vitamins, and antioxidants, diatoms have become valuable sources of food supplements for humans [29]. Moreover, they can synthesize large amounts of polyunsaturated fatty acids, which are bioactive substances proven to promote human health (e.g., decrease in frequency of cardiovascular diseases and cancers) and growth in animals [19,30,31]. Additionally, the major carotenoid of diatoms, the brown-colored fucoxanthin, is used as an antioxidant, anti-inflammatory, anti-diabetes and anti-cancer drug [32,33], as well as for its protective effect on liver, eyes, blood vessels, skin, and lungs [32,34]. Anti-inflammatory and immunostimulating activities of diatoms polysaccharides such as laminarin have also been reported as effective in various fish species [35,36,37]. Additionally, other polysaccharides such as chrysolaminarin from the diatom *Chaetoceros muelleri* have been shown to be promising candidates as immuno-stimulatory food additives in aquaculture [38]. Chrysolaminarin isolated from the diatom *Synedra acus* shows anti-tumor activity by inhibiting the proliferation of human colon cancer cells and colony formation [39]. Recently, polysaccharides from algae (including diatoms) have attracted interest in the cosmetic industry: some sulfated polysaccharides have already been tested to prevent the accumulation and the activity of free radicals and reactive chemical species, therefore acting as protective systems against oxidative stress [40]. The use of diatoms is thus likely to expand in the future. Additionally, the diatom *P. tricornutum* has recently been evaluated as a potential solar-fueled expression system to produce bioplastics [41] and biopharmaceuticals. In the biopharmaceutical field, diatoms have successfully been used to produce functional monoclonal human IgG antibodies directed against the hepatitis B virus surface antigen [42,43]. Understanding post-translational modifications (including glycosylation processing) in diatoms is fundamental, because they determine the critical quality attributes that can influence folding, half-life, activity, and immunogenicity of biopharmaceuticals [44,45].

Glycoconjugates, such as glycans and polysaccharides, are assembled and modified within the endomembrane system [46]. Their synthesis involves three steps, the first being the formation of activated nucleotide sugars, such as NDP-sugars or NMP-sugars within the cytosol [47]. Then, the nucleotide sugars are actively transported to the endoplasmic reticulum (ER) and Golgi apparatus where they serve as donor substrates for glycosyltransferases (GT) that transfer a specific sugar from its activated nucleotide form to a specific acceptor leading to the extension of the glycoconjugates.

In this review, in regard to the capability of diatoms to synthesize glycoconjugates, we focus on the composition, structure and properties of diatom polysaccharides—whether they be intracellular, cell wall-bound or secreted in the culture medium—and on the structure and biosynthesis of *N*-glycans attached to proteins.

## 2. Monosaccharide Composition and Structures of Polysaccharides in Diatoms

**Figure 2:**
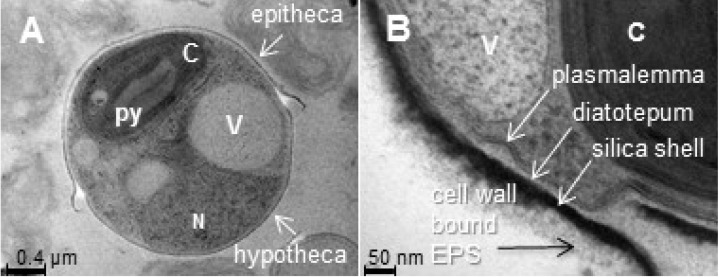
Ultrastructural organization of a diatom cell: transmission electron micrographs of *Phaeodactylum tricornutum* oval morphotype. The cells were embedded in LRW resin with 0.5% uranyl acetate in a methanol/Reynold’s lead citrate solution. (**A**) general overview of a *P. tricornutum* oval cell. Scale bar = 0.4 µm; (**B**) zoom of the cell wall. Scale bar = 50 nm. N: nucleus; V: vacuole; C: chloroplast; py: pyrenoid; EPS: exopolysaccharides.

Given the thousands of diatom species, with their large variety of forms, symmetry and cell wall shapes, the monosaccharide composition and structure of diatom glycoconjugates are likely to be highly specific. Diatoms are usually described as single cells with a protoplast embedded in a frustule—the name for the diatom cell wall—composed of two overlapping valves or thecae: the larger, upper epitheca and the smaller, lower hypotheca (Figure 2A). The frustule is composed of three successive layers: (1) the inner-most, organic layer, called the diatotepum, is in contact with the plasmalemma; (2) a mineral, silicified shell that contains organic matter; and finally (3) an external organic coat that is trapped in secreted mucilage, that we call here “cell wall-bound exopolysaccharides (EPSs)” (Figure 2B). Numerous studies have characterized the monosaccharide composition of cell wall polysaccharides, intracellular food storage polymers, and extracellular mucilage, but results must be carefully interpreted according to the extraction techniques used, which depend on the solubility of the respective components [48,49,50,51,52,53].

### 2.1. Insoluble Polysaccharides in Diatoms

#### 2.1.1. Frustules, or Cell Wall Polysaccharides

In diatoms, cell wall silica is associated with organic matter, composed mainly of proteins [54,55,56], polyamines [57], and polysaccharides [58]. Three families of proteins have been isolated from *C. fusiformis* cell walls: frustulins, pleuralins, and silaffins (see reference [59] for a review). Long polyamine chains, together with silaffins, are likely involved in frustule biosynthesis. However, the location and role of each component involved in frustule biosynthesis are not well-understood. Experimental studies on the chemical composition of the organic matter in diatom cell walls have demonstrated that polysaccharide content dominates that of proteins and lipids [60], although X-ray photoelectron spectroscopy measurements of cell surface components in *T. pseudonana* appear to show that polysaccharides are not predominant [61]. To characterize and localize these polysaccharides, accurate sequential extraction is necessary for at least three reasons. First, depending on their composition and structure, polysaccharides have various water-solubility properties, which in turn vary with temperature and chemical treatments. Second, cell wall polysaccharides may be more or less tightly bound either together, to silica, or to other insoluble components of the frustule. Third, a secreted or an extracted substance is not necessarily water-soluble once outside the cell; thus an insoluble extracted polysaccharide is not necessarily a cell wall component.

Based on typical sequential extractions performed either on live or mechanically disrupted cells, we reviewed the monosaccharide composition reported in the three final fractions: (1) hot alkali soluble fraction; (2) hot alkali insoluble fraction; and (3) residual material that makes up the insoluble organic cell wall fraction (see Table 1). Different monosaccharide profiles have been observed in alkali soluble fractions: fucose dominates in *Thalassiosira gravida* and *Corethron hystrix*; rhamnose is mostly encountered in *Chaetoceros affinis* [62], galactose in *Thalassiosira weissflogii* [63] and mannose in *P. tricornutum*. Fractions with high levels of ribose were attributed to the cross-contamination of the extracts from intracellular content [50,62]. In the alkali insoluble fraction, the monosaccharide composition profile is not that different from that found in insoluble organic cell walls in which mannose is preponderant. Insoluble organic cell walls, as summarized over 19 taxa in Table 1, and first shown by Coombs and Volcani [64], and reviewed by Hoagland *et al.* [48], contain fucose, galactose, glucose, mannose, xylose, glucuronic acid residues and, to a much lesser extent, rhamnose and arabinose. Although mannose is obviously the most abundant monosaccharide, fucose is dominant in *Nitzschia brevirostris*, whereas glucose predominates in *Melosira granulata* and *Cyclotella stelligera* [55], *Nitzschia curvilineata* and *Amphora salina* [53]. It is difficult to determine the abundance of other monosaccharide components due to their heterogeneous representation.

**Figure 3:**
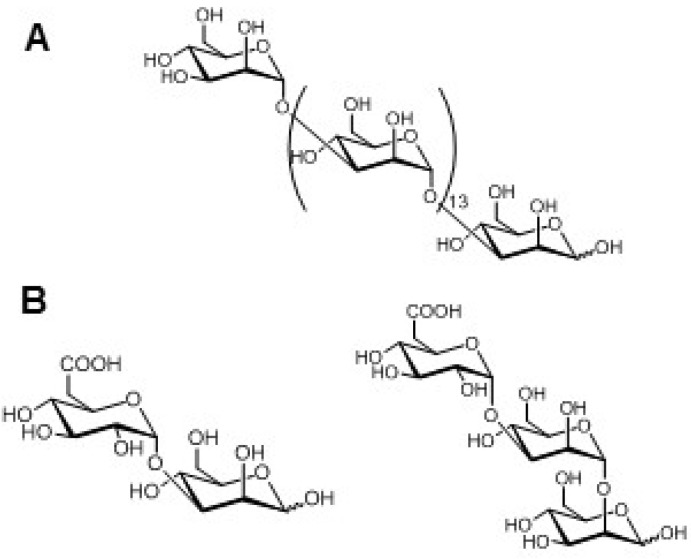
Drawings of hypothetical structures of oligosaccharides found in insoluble cell wall polysaccharides after mild acid hydrolysis of *Phaeodactylum tricornutum* cell wall extracts. (**A**) 1,3-linked mannopyranose chains; (**B**) oligosaccharide fragments. Although hypothetical alpha-linkages are shown here, there is no clear evidence for either alpha- or beta-linkages [65].

The best studied frustule polysaccharides have been extracted from *P. tricornutum*. Based on successive alkali extraction, deproteination, chromatographic separation, Percival and co-workers extracted a cell wall polysaccharide from *P. tricornutum* [65] that is mainly composed of mannose, glucuronic acid residues and sulfate groups. Mild acid hydrolysis of the polysaccharides combined with chemical analysis of the oligosaccharide fragments revealed moieties that should be present in the overall polysaccharide structure. Blocks of 3-linked mannose have been identified and are assumed to form the backbone of the polysaccharides (Figure 3A). Substitution at position 2 of the mannose of the main chain with di- and trisaccharides composed of mannose and glucuronic acid (Figure 3B), or with sulfate groups have also been described in *Pinnularia viridis* [52] as well as in *P. tricornutum* [66]. However, the detailed configuration of the linkage between residues, as well as the size and distribution of the ramification are still unknown. The cell wall monosaccharide composition of several diatom species includes high amounts of mannose and glucuronic acid and low, but more variable amounts of fucose and xylose. According to these results, the glucuronomannan described in *P. tricornutum* may be synthesized more generally by other diatoms [52,66,67]. Environmental conditions have been shown to influence the monosaccharide composition of cell wall polysaccharides, thus affecting their respective structures as illustrated in *P. tricornutum* [66]. Variations in culture conditions, such as phosphate limitation in the culture medium, an increase in salinity, switching culture from liquid to solid medium—all considered as stress conditions—may cause the observed enrichment in rhamnose, uronic acid, sulfate, and *O*-methylated sugars in the insoluble polymeric fraction. Such variation in monosaccharide composition is assumed to modify polysaccharide structures, enabling cells to adapt to environmental changes. Therefore, the effects of culture conditions on the monosaccharide composition of glycoconjugates must be considered when comparing experimental results.

**Table 1:** Summary of the monosaccharide composition of diatom extracts: alkali soluble fraction, alkali insoluble fraction, and insoluble organic cell wall residues. Values are expressed in mol% of total monosaccharides detected in extracts. Horizontal sums of values lower than 100% indicate that some monosaccharides were not clearly identified in the corresponding study.

Monosaccharide		Ara	Fuc	Gal	Glc	Man	Rha	Rib	Xyl	3-O-MeFuc	2-MeGal	3/4-MeGal	GalA	GlcA	2-MeGlcA	ManA	2-MeRha	3-MeRha	2,3-diMeRha	3-MeXyl	4-MeXyl	Unknown	GlcNac
*^a^* *Chaeotoceros affinis*	Alkali soluble fraction		11	18	2	6	52	—	7														
*^a^* *Chaeotoceros curvisetus*			32	31	1	6	16	10	4														
*^a^* *Chaeotoceros decipiens*			4	7	6	32	25	4	10														
*^a^* *Chaeotoceros debilis*			11	23	6	—	22	23	15														
*^a^* *Chaeotoceros socialis*			18	16	3	9	23	11	8														
*^a^* *Thalassiosira gravida*			43	12	4	7	—	27	7														
*^a^* *Corethron hystrix*			60	9	11	9	1	8	2														
*^b^* *Thalassiosira weissflogii*		1.94	6.98	36	19.5	17.9	4	3.91	7.31														
*^c^* *Phaeodactylum tricornutum*		0.3	2.3	1.7	2.4	55.6	11.7	—	5.4	nd	tr	—	1.9	6.7		12	nd	nd	—	nd	nd		
*^d^* *Phaeodactylum tricornutum* O		7	3	8	18	28	9	1	8			tr					tr			tr			
*^d^* *Phaeodactylum tricornutum* F			2	6	11	45	7		8			2		10									2
*^e^* *Stauroneis amphioxys*	Alkali insoluble fraction	—	1	9	7	50	2		2	2			nd	28	nd		nd	2	nd	—	—		
*^d^* *Phaeodactylum tricornutum* O			1	5	12	72	1	tr	1														
*^d^* *Phaeodactylum tricornutum* F		4	1	12	10	66	2	tr	4			1											
*^f^* *Nitzschia frustulum*	Insoluble organic cell walls	—	14	14	tr	32	5	—	tr													35	
*^f^* *Nitzschia angularis*		—	64	4	14	11	tr	—	tr													7	
*^f^* *Asterionella socialis*		—	tr	5	tr	22	tr	—	tr													78	
*^f^ Cylindrotheca fusiformis*		—	tr	10	tr	12	tr	—	tr													78	
*^g^* *Navicula pelliculosa*		—	9.2	9.9	25.9	48.5	3.8	—	3.1														
*^g^* *Melosira nummuloides*		0.3	25.6	3.7	0.9	56.8	1.8	—	10.9														
*^g^* *Melosira granulata*	Insoluble organic cell walls	0.6	0.8	5.4	46.7	6.5	1.2	—	38.9															
*^g^* *Cyclotella stelligera*		2	0.6	22.4	43.3	13.6	0	—	18															
*^g^* *Cyclotella cryptica*		—	12.2	12.2	13	37.2	7.4	—	17.8															
*^g^* *Nitzschia brevirostris Hust.*		1.7	42.5	9.5	14.6	20.2	2.4	4.7	4.3															
*^h^* *Pinnularia viridis*		tr	1.5	7	13	54	9.5		11				—	—	—		2	1	1	—	—			
*^h^* *Craspedostauros australis*		tr	tr	2	5	69	2		4				2	2	12		—	—	—	2	—			
*^h^* *Thalassiosira pseudonana*		1	2	10	6	65	tr		5				—	10	—		—	tr	—	—	1			
*^h^* *Nitzschia navis-varingica*		2	1	3	7	64	2		1				tr	15	—		—	5	—	—	—			
*^i^* *Coscinodiscus radiatus*				tr	4.7	80.1	1.5		1.4				tr	12.4										
*^i^* *Nitzschia curvilineata*				6.5	45.6	40.3	1.7		4.4				—	1.4										
*^i^* *Amphora salina*				2.7	47.7	41.5	1.5		1.3				tr	5.5										
*^i^* *Triceratium dubium*				tr	6.4	67.4	tr		6.3				5.6	1.5									12.8	
*^d^* *Phaeodactylum tricornutum* O			3	3	11	61	12		2			4												
*^d^* *Phaeodactylum tricornutum* F		3	1	10	25	47	3	tr	6			2												

tr, trace (<0.8% mol); —, not detected; nd, not determined. Bright yellow: major compound, green: second-most major compound. ^a^ [62]; ^b^ [63]; ^c^ [66]; ^d^ [67]; ^e^ [68]; ^f^ [69]; ^g^ [55]; ^h^ [52]; ^i^ [53]. Morphotypes of *Phaeodactylum tricornutum*: F, fusiform; O, oval. Ara, arabinose; Fuc, fucose; Gal, galactose; GalA, galacturonic acid; Glc, glucose; GlcA, glucuronic acid; GlcNAc, *N*-acetylglucosamine; Man, mannose; ManA, mannuronic acid; Me, methyl group; Rha, rhamnose; Rib, ribose; Xyl, xylose.

#### 2.1.2. Chitinous Spines

**Figure 4:**
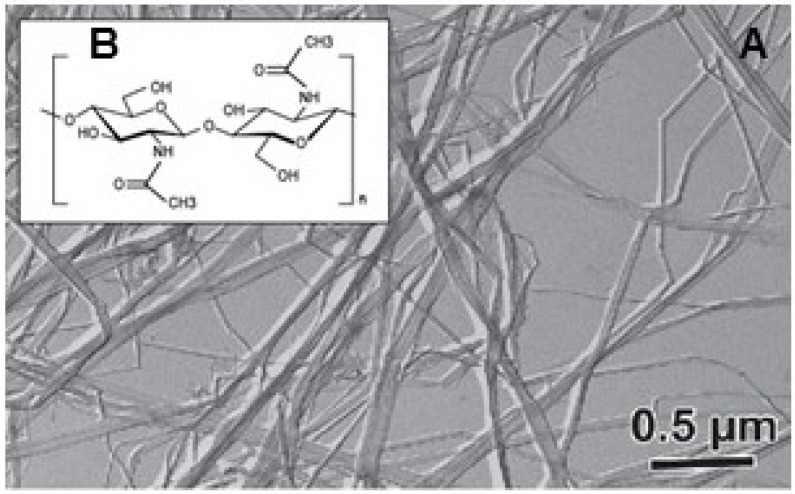
β-chitin fibers of *Thalassiosira* sp. (**A**) Transmission electron micrograph shadowed with tanta-lum/tungsten (Ta/W). (**B**): *N*-acetylglucosamine sequence of the chemical structure of chitin. The image was recorded and kindly provided by Dr. H. Chanzy, CERMAV-CNRS, France.

*Cyclotella* and *Thalassiosira* species produce stiff and highly crystalline fibers of chitin (poly-*N*-acetyl-d-glucosamine), as demonstrated using chemical, crystallographic, and enzymatic methods [70,71,72]. Due to high crystallinity (chitin is probably the most crystalline polysaccharide material on earth); the crystal structure of chitin fibers has been resolved at the molecular level [73,74]. Diatoms secrete β-chitin (Figure 4), which has a crystalline structure similar to that described in worms [75,76,77], but different from that of arthropods, crustaceans, and fungi, which all synthesize α-chitin. α-Chitin shows anti-parallel chain packing, whereas β-chitin polymer chains show parallel packing, meaning that reducing ends all point out in the same direction. In diatoms, chitin fibers are excreted through specialized pores within the thecae called fultoportulae. Cross-sections examined under transmission electron microscopy show invaginations of the plasma membrane at the site of chitin polymerization [78,79,80]. Similar secretion systems have been reported for the giant tube worm *Riftia pachyptila* [81,82]. Crystallographic analyses of chitin fibers bound to thecae demonstrate that chitin polymerization occurs by elongation at the non-reducing end, consistent with the reducing chain end being the furthest from the biosynthesis site [83,84].

Genes encoding chitin synthase were discovered in the *T.* *pseudonana* genome. Homologous genes, but no chitin fibers, have been described in *Skeletonema costatum*, *Chaetoceros socialis*, *Lithodesmium undulatum* and *P. tricornutum*, suggesting a common origin of chitin synthase in diatoms, but also indicating potential occurrence of yet undescribed chitin [85]. Chitin occurs in the silica frustule of *T. pseudonana* [86] and is probably an underestimated component of diatom cell walls in general. Inhibition of chitin synthase or chitin crystallization mainly increases the sedimentation rate of diatoms. This effect suggests that chitin fibers are involved in the buoyancy of the dense siliceous diatom cells. The importance of chitin fibers in diatoms has also been highlighted: the chitin content accounts for an estimated 30% of the organic carbon pool in *Cyclotella* species [87].

### 2.2. Soluble Polysaccharides in Diatoms

#### 2.2.1. Food Storage Polysaccharides

The food storage polysaccharide in diatoms is a β(1,3) glucan, also called chrysolaminarin because it resembles the β(1,3) glucan found in chrysophyte algae [88]. This particular polysaccharide has been localized in the vacuole using aniline blue dye [89] and anti-β(1,3) glucan antibodies [51]. Vacuolar accumulation is enhanced during photosynthesis and is mobilized in the dark. β(1,3) glucan content can reach up to 20%–30% of dry matter during the exponential growth phase of the diatom [90] and up to 80% during the stationary phase.

**Figure 5:**
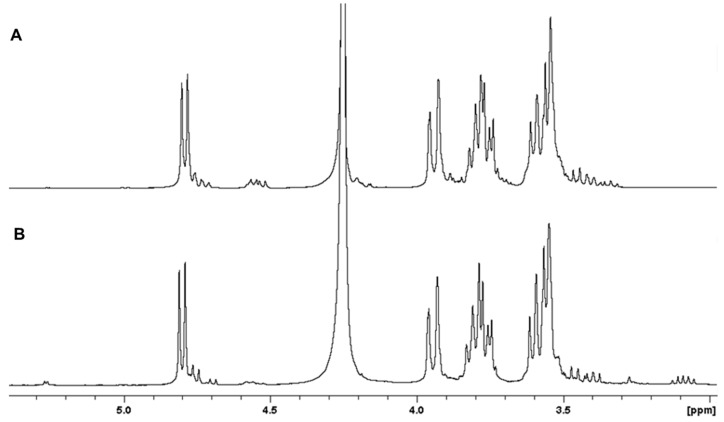
Structural analysis of β(1,3) glucan, a food storage polysaccharide. ^1^H NMR spectra of (**A**) laminarin from *Saccharina latissima* and (**B**) β(1,3) glucan (chrysolaminarin) extracted from *Phaeodactylum tricornutum* (400 MHz, 353 K). Chrysolaminarin contains fewer β(1,6) branching signals (4.5–4.6 ppm) than laminarin. The slightly higher reducing end signal at 5.26 ppm (α-anomer) in the chrysolaminarin spectrum can be attributed to a lower molecular weight or the absence of a mannitol residue at the reducing end.

Treating diatom cells with hot or boiling water is often sufficient to extract chrysolaminarin as the main polysaccharide component. Mild acid hydrolysis and freeze-drying also help cell wall disruption. Chrysolaminarin is insoluble in organic solvents and can be easily recovered by precipitation in alcohol or acetone [91,92,93]. The structure of diatom β(1,3) glucans was first described from a mixed bloom of freshwater diatom species that included *Nitzschia sigmoidea*, *Cymatopleura solea*, *Pinnularia* sp. and *Melosira varians* [91]. Since then, chrysolaminarin structures from several diatom species (*Skeletonema*, *Phaeodactylum*, *Chaetoceros*, *Thalassiosira*) have been studied using chemical analysis and spectroscopic methods such as NMR (Figure 5). The NMR spectra given in Figure 5 show high degrees of similarities between the β(1,3) glucan extracted from *Saccharina latissima* (Figure 5A) and from *P. tricornutum* (Figure 5B) , with the exception of fewer β(1,6) branching signals for chrysolamaninarin and a slightly higher reducing-end signal. The *P. tricornutum* β(1,3) glucan may have lower molecular weight or lack a mannitol residue at the reducing end.

Based on the numerous studies, a general picture of diatom chrysolaminarin structure has emerged. It is usually composed of a β(1,3) glucan backbone chain ramified with β(1,6) glucose and sometimes with β(1,2) glucose. The length of the backbone chain and the degree of ramification vary with the diatom species (Table 2). During the growth phase of *T. weissflogii* and *C. muelleri*, the structure of chrysolaminarin does not change noticeably, suggesting that culture conditions do not influence the chrysolaminarin structure [94].

**Table 2:** Overview of the structural features of diatom chrysolaminarins. For comparison, *Laminaria digitata* laminarin has a degree of polymerization (DP) of 20–30 residues and a degree of branching (DB) of 0.05, [95]. Yield extraction of chrysolaminarin is expressed in % of diatom dry weight.

Species	Mw/DP	Branching	Yield% (*w*/*w*)	Reference
*Phaeodactylum tricornutum*	nd	Some β(1,6) branching	14%	[92]
*Skeletonema costatum*	6–13 kDa	Some β(1,6) and β(1,2) branching	32%	[96]
*Stauroneis amphioxys*	4 kDa, DP ~24	Some β(1,6) and β(1,2) branching	nd	[97]
*Achnanthes longipes*	nd	Small degree of β(1,6) and β(1,2) branching	nd	[98]
*Pinnularia viridis*	>10 kDa	Small degree of β(1,6) branching	nd	[99]
*Aulacoseira baicalensis*	3–5 kDa	nd	0.9%	[93]
*Stephanodiscus meyerii*	40 kDa	β(1,6)/β(1,3) DB 0.053 ^a^	0.5%	
*Stephanodiscus meyerii*	2–6 kDa	β(1,6)/β(1,3) DB 0.25 ^a^	0.4%	
*Aulacoseira baicalensis*	nd	β(1,6)/β(1,3) DB 0.11 ^a^ Mannitol detected	0.6%	
*Chaetoceros muelleri*	DP 22–24	β(1,6)/β(1,3) DB 0.006–0.009	nd	[94]
*Thalassiosira weissflogii*	DP 5–13	No branching	nd	
*Chaetoceros debilis*	4.9 kDa, DP 30	β(1,6) 37% of total residue	10%	[100]

nd: not determined; ^a^ calculated based on published data.

Based on the biological activity of β(1,3) glucans (see Section 1), laminarin is currently marketed for its ability to stimulate macrophages leading to immuno-stimulatory, anti-tumor and wound-healing activities [101].

#### 2.2.2. Exopolysaccharides

Diatoms synthesize extracellular mucilage, which mainly consists of complex heteroglycans. Although EPS usually refers to exopolysaccharides, in its broad sense it includes all extracellular polymeric substances, which have high carbohydrate contents [99,102,103], and can even be used to mean any macromolecule secreted from the plasmalemma (see review by Hoagland *et al.*, reference [48]). EPSs have been described in many forms, such as stalks, tubes, apical pads, adhering films, fibrils, and cell coatings, which imply that EPS components have a wide variety of morphologies, ranging from highly crystalline rigid fibrils to highly hydrated mucilaginous capsules, and including polymers that are tightly bound to or integrated in the cell wall. In this section, we focus only on soluble EPSs.

Excretion of EPSs by diatoms provides a food source for heterotrophic organisms and affects the erodibility of biofilms [102,103,104]. EPS production rates and their monosaccharide compositions differ according to the growth phase and the physiological status of the cells [68,103,104,105]. EPS secretion depends on environmental conditions such as nutrient availability, daily fluctuations, irradiance, and even metal toxicity [106,107,108,109,110]. Studies have shown that nitrogen (*N*) and phosphate (*P*) limitations affect the production rate of EPSs as well as their monosaccharide compositions. For example, *N* or *P* limitation have been shown to stimulate EPS production in various diatoms [66,108,110]. Under *P*-limited conditions in *C. fusiformis*, monosaccharide composition shows an increase in galactose and a decrease in glucose, whereas the composition of the remaining monosaccharides is almost unaffected [108]. EPS production also increases under *P*-depleted conditions in other diatom species [109] with reduced glucose content in *Cylindrotheca closterium*. Likewise, fucose and rhamnose appear to be involved in adhesion, either by enrichment in some biofilm EPS structures with those residues, or by modification of the linkage types of those residues [67,111]. Mass spectrometry on *T. pseudonana* EPSs has shown that the degree of polymerization and the distribution of EPSs vary in response to nutrient depletion and different nutrient sources [110].

As in cell wall polysaccharides, the monosaccharide composition of EPSs can vary drastically depending on the extraction method [112]. However, diatom EPSs have two general compositional features: (1) they consist of heteropolysaccharides that can be sulfated and (2) they contain rhamnose, fucose, galactose, glucose, mannose, xylose and/or uronic acids as well as some arabinose in lower proportions (Table 3). Furthermore, diatom EPSs have high proportions of methylpentoses compared with intracellular soluble and cell wall polysaccharides. To date, no complete fine structure has been resolved for diatom EPSs and only a few studies report data on linkages. However, the available data show a large diversity of linkages found in diatom EPSs, with many glycosyl residues being typical of branched structures (Table 3).

**Table 3:** Structural characteristics of some exopolysaccharides (EPSs) in diatoms: monosaccharide composition, sulfate substitution, linkages. The data are non-exhaustive and only include soluble EPSs recovered from culture media.

Species	Monosaccharides	Sulfate ^a^ (wt%)	Linkages	Reference
*Amphora* sp. F1	GlcA(1.6)/Gal(1.1)/Fuc(1) ^b^	9.7	nd ^c^	[113]
*Amphora* sp. F2	GlcA(2.8)/Fuc(1)/Gal(0.8)	18.2	nd	
*Amphora holsatica*	UA/Rha/Fuc/Glc/Xyl/Ara ^d^	nd	nd	[114]
*Amphora rostrata*	Fuc(41)/Gal(32)/UA(23)/Man(9)/Rha(8)	10	nd	[115]
*Asterionella socialis* ^e^	Rha(70)/Man(7)/2 Unk(23)	nd	nd	[69]
*Chaetoceros affinis* ^e^	Fuc(39)/Rha(35)/Gal(26)	8.7	t-Fuc ^f^, 2,3-Fuc, 3,4-Fuc, 3-Fuc/2-Rha, t-Rha, 3-Rha, 3,4-Rha/3-Gal, t-Gal, 4-Gal _f_	[116,117]
*Chaetoceros curvisetus* ^e^	Fuc(35)/Gal(10)/Rha(3)	7	2-Fuc _f_, t-Fuc _f_, 2,3-Fuc, 3-Fuc, 2,3-Fuc _f_, 3,4-Fuc, 3,5-Fuc _f_/3-Gal, 2,3-Gal, t-Gal/2-Rha, t-Rha	[118]
*Chaetoceros debilis* ^b^	Fuc(30)/Gal(29)/Rha(17)/Man(10)/Xyl(9)/Glc(5)	nd	nd	[62]
*Chaetoceros decipiens* ^e^	Rha(34)/Fuc(32)/Gal(17)/Man(7)/Xyl(5)/Glc(5)	nd	nd	
*Coscinodiscus nobilis* ^b^	Fuc(34)/Man(19)/Glc(16)/Rha(15)/GlcA(9)/Xyl(6)	16.7	3-Fuc/6-Man/3-Glc/2-Rha/t-Xyl with Fuc and Rha branched or sulfated	[119]
*Cylindrotheca closterium*	Xyl(46)/Glc(23)/Rha(15)/Gal(12)/Man(4)/UA(5)	0	nd	[107]
*Cylindrotheca fusiformis*	Gal(38)/Glc(26)/Xyl(13)/Rha(13)/UA(7)/Man(5)	31	nd	[108]
*Cyclotella nana* ^e^	Rha(33)/Gal(14)/Glc(11)/Man(10)/Rib(8)/Xyl(7)/2 Unk(17)/GlcA(?)	nd	nd	[69]
*Melosira nummuloides*	UA/Rha/Fuc/Glc/Xyl/Ara/Gal ^d^	nd	nd	[114]
*Navicula directa*	UA/Rha/Fuc/Glc/Ara/Gal/Xyl ^d^	nd	nd	
*Navicula incerta* ^e^	Rha(33)/Fuc(20)/Man(10)/Xyl(9)/Gal(8)/GlcA(?)/3 Unk(20)	nd	nd	[69]
*Navicula salinarum*	Glc(41)/Xyl(20)/Gal(19)/Man(14)/Rha(5)/UA(21)	6.3	nd	[107]
*Navicula subinflata*	Glc(94)/UA(9)	9.6	nd	[120]
*Nitzschia angularis* ^e^	Rha(20)/Gal(17)/Fuc(16)/Ara(8)/Man(7)/Xyl(7)/GlcA(?)/2 Unk(25)/	nd	nd	[69]
*Nitzschia frustulum* ^e^	Man(34)/Rha(24)/Gal(8)/GlcA(?)/2 Unk(34)	9	nd	
*Pinnularia viridis*	Rha(29)/Gal(23.5)/Xyl(17)/Glc(7.5)/Man(6.5)/Fuc(6)	nd	3-Rha, 3,4-Rha, 2,3-Rha, 2-Rha, t-Rha/ 3-Gal, 3,6-Gal/t-Xyl, 2,4-Xyl, 4-Xyl/4-Glc/4-Man, t-Man/t-Fuc, 2-Fuc	[99]
*Thalassiosira* sp. F1	Man(51)/Rha(19)/Fuc(8)/Xyl(6)	nd	t-Man, 4,6-Man, 4-Man/3-Rha, 2-Rha/3-Fuc, t-Fuc/t-Xyl, 2-Xyl	[121]
*Thalassiosira* sp. F2	Man(57)/Xyl(19)/GlcA(6)/GalA(5)	nd	4-Man, t-Man, 2-Man/4-Xyl, t-Xyl/t-GlcA/t-GalA	

^a^ Sulfate percentages represent % weight of isolated polymers; ^b^ Only the sugars with contents of >5% are reported. Ara, arabinose; Fuc, fucose; Gal, galactose; Glc, glucose; GlcA, glucuronic acid; Man, mannose; Rha, rhamnose; Rib, ribose; UA, uronic acid; Unk, unknown; Xyl, xylose. Numbers in brackets following abbreviations give relative proportions of monosaccharide residues expressed as mol%, wt%, molar ratio, *etc.*, as reported. The sugars are ordered from high to low percentages for each species; ^c^ nd, not determined; ^d^ The ratio varies with hydrolysis conditions; ^e^ Data also available in the review [48]; ^f^ Glycosyl linkages expressed as the position(s) of substitution in addition to C-1 (t-Fuc, terminal fucosyl; 3-Rha, 3-rhamnosyl); Subscript “f” following sugar abbreviation indicates furanose form.

## 3. Structures and Biosynthesis of Protein *N*-Glycans in Diatoms

**Figure 6:**
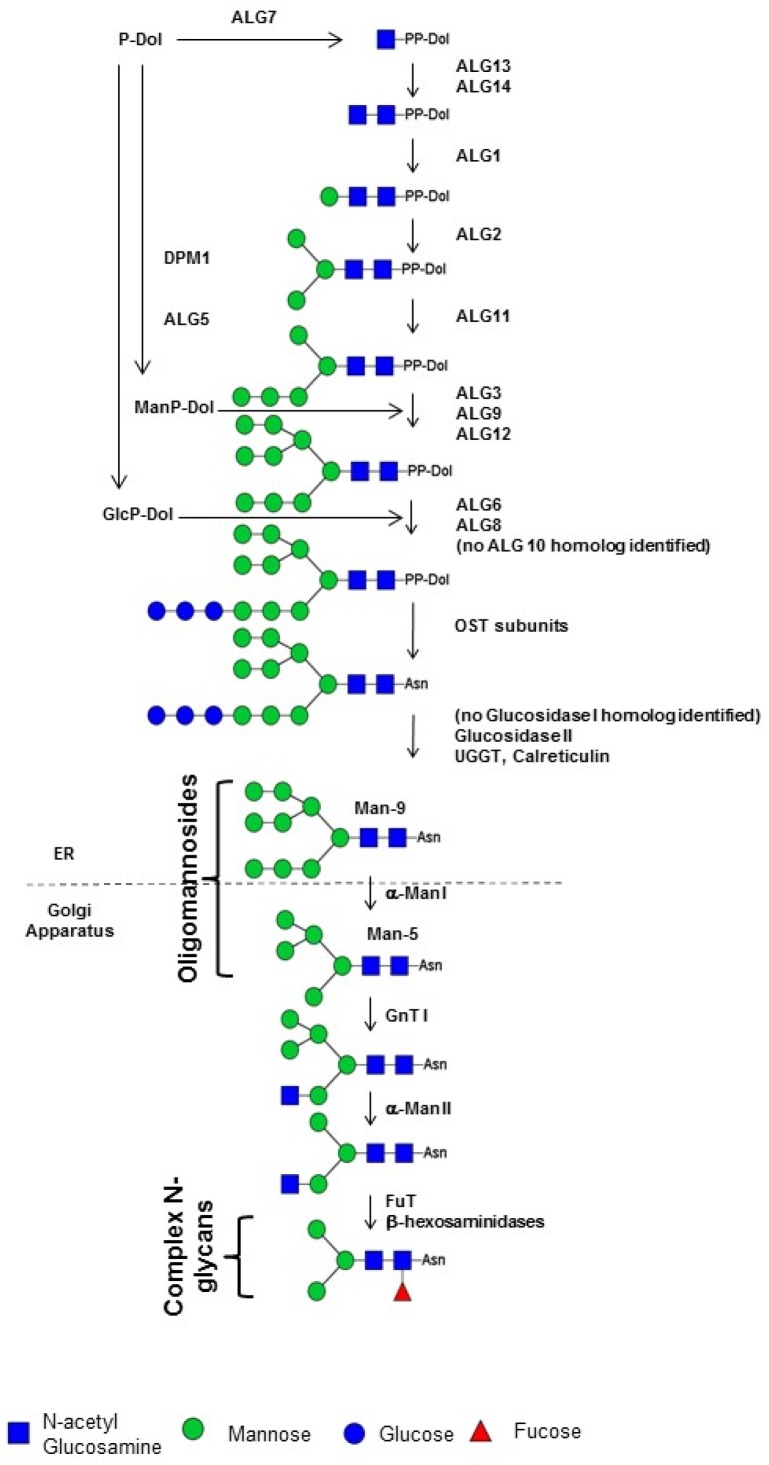
Proposed *N*-glycosylation pathway in *Phaeodactylum tricornutum*. Sequences predicted in the *P. tricornutum* genome are shown in bold*. ALG10* and glucosidase I genes have not been identified so far. The *N*-glycan structures presented in this figure are as given in Varki *et al.* [121]. ER: endoplasmic reticulum; DPM1: dolichol-phosphate mannosyl transferase; ALG: asparagine-linked glycosylation; PP-Dol: pyrophosphate dolichol; P-Dol: dolichol phosphate; OST: oligosaccharyl transferase; Asn: asparagine; UGGT: UDP-glucose glycoprotein glucosyl transferase; GnT: N-acetylglucosaminyl transferase; α-Man: α-Mannosidase; FuT: fucosyl transferase; Man-5 to Man-9: oligomannoside bearing 5 to 9 mannose residues.

*N*-glycosylation is a major co- and post-translational modification of proteins in eukaryotes occurring in both the ER and the Golgi apparatus (Figure 6, [122]). In this process, a lipid-linked oligosaccharide composed of three glucose (Glc), nine mannose (Man) and two GlcNAc residues (Glc_3_Man_9_GlcNAc_2_) is first assembled by the stepwise addition of monosaccharides on a dolicholpyrophosphate on the cytosolic side of and then in the lumen of the ER [123]. This oligosaccharide precursor is then transferred by the oligosaccharyl transferase (OST) complex onto the asparagine residues of consensus Asn-X-Ser/Thr sequences of a protein [123]. In 3.5% of studied cases, other sequences such as Asn-X-Cys, Asn-X-Val are glycosylated in endogenous or recombinant proteins produced in mammals or plant cells [124,125,126]. The glycoprotein is deglucosylated by α-glucosidases I and II and then reglucosylated by an uridine diphosphate (UDP)-glucose glycoprotein glucosyl transferase (UGGT) to ensure proper folding of the nascent protein through its interaction with ER-resident chaperones, such as calnexin and calreticulin [127]. These ER events are conserved in eukaryotes because they are crucial for efficient protein folding [127]. Bioinformatic analyses demonstrate that most of the genes encoding enzymes involved in the biosynthesis of the dolicholpyrophosphate-linked oligosaccharide, named asparagine-linked glycosylation (ALG) [128], are predicted in the genomes of diatoms (*P. tricornutum* [129], *T. pseudonana* [130], *Fragilariopsis cylindrus* [131] and *Aureococcus anophagereffens* [132]) (Figure 6), [133,134,135]. The only exception is ALG 10, an α(1,2)-glucosyl transferase responsible for the transfer of the terminal Glc residue of the triglucosyl extension of the *N*-glycan precursor, for which no homology has been found (Figure 6) [133,135]. In addition to ALG genes, genes encoding subunits of the oligosaccharyl transferase have also been identified in diatom genomes, especially in *P. tricornutum* (Figure 6), in which α-glucosidase II (but not α-glucosidase I) as well as ER-resident UGGT and chaperones such as calreticulin are also predicted (Figure 6) [133,135]. These proteins are key elements of the quality control of proteins occurring in the ER. Large oligomannosides, with sizes of up to Man_9_GlcNAc_2_, have been found in *P. tricornutum* glycoproteins [133], suggesting that the synthesis of the oligosaccharide precursor and the quality control of secreted proteins may occur in a similar manner as that observed in other eukaryotes. However, in the ER, α-glucosidase I appears to remove the terminal α(1,2)-glucosyl transferase that is transferred by ALG10. Absence of ALG 10 and α-glucosidase I genes in *P. tricornutum* suggests that the *N*-glycan precursor is not fully glucosylated in diatoms into Glc_3_Man_9_GlcNAc_2_ (Figure 6) [133,135].

In contrast to ER events, evolutionary adaptation of *N*-glycan processing in the Golgi apparatus has given rise to a variety of organism-specific complex structures [136]. First, α-mannosidases (α-Man I) degrade the oligosaccharide precursor into oligomannosides ranging from Man_9_GlcNAc_2_ to Man_5_GlcNAc_2_ (Man-9 to Man-5) (Figure 6). *N*-acetylglucosaminyl transferase I (GnT I) then transfers a first *N*-acetylglucosaminyl (GlcNAc) residue onto Man-5 and initiates the synthesis of a large variety of structurally different complex-type *N*-glycans. This processing continues with the removal of two mannosyl residues and then decoration of the *N*-glycans by the action of a specific repertoire of glycosyl transferases such as α-fucosyl transferases (FuT). Therefore, mature proteins leaving the secretory pathway carry organism-specific complex *N*-glycans allowing the protein to acquire a set of glycan-mediated biological functions [137,138]. Searches in diatom genomes for candidate genes encoding Golgi glycosidases and glycosyl transferases involved in *N*-glycan processing have led to the identification of α-Man I, GnT I and a FuT (putative α(1,3)-FuT) candidates [133,134], (Figure 6). The GnT I gene predicted in the *P. tricornutum* genome has been demonstrated to encode an active functional enzyme able to restore the maturation of *N*-linked glycans into complex-type *N*-glycans in the CHO Lec1 mutant which is affected in its endogenous GnT I [133]. Moreover, structural analysis of glycans *N*-linked to proteins secreted by the diatom *P. tricornutum* indicate that these oligosaccharides are processed through a GnT I-dependent pathway into partially fucosylated Man_3_GlcNAc_2_ (Figure 6) [133]. This truncated and fucosylated *N*-linked glycan likely results from the trimming of two mannose residues from Man-5 by an α-Man II and then transfer of an α(1,3)-fucose residue [133]. Later, the terminal GlcNAc introduced by the Golgi *P. tricornutum* GnT I are probably eliminated by β-hexosaminidases, as previously described in land plants and insects [139,140]. Two putative β-hexosaminidases have already been identified in *P. tricornutum* [133].

In addition, diatoms may have glycoproteins bearing *O*-glycans. For example, Swift and Wheeler showed that some proteins associated with the silica shell, called frustule-associated components (FACs), can be easily extracted from cell walls with an EDTA-based treatment [56]. These fractions can contain glycoproteins. In *C. fusiformis*, EDTA-soluble proteins [141], called frustulins, bear a glycan moiety composed of rhamnose (25%), galactose (20%), xylose (20%), glucose (2%), and mannose (1%). Although first described as proteoglycans [142], glycoproteins have also been isolated [99] in *Craspedosauros australis* extracted with urea and exhibit a xylose-rich composition (41%), with lower amounts of galactose, rhamnose, and mannose (14%, 12%, 10%, respectively). More work needs to be done to fully characterize the glycan structures of such glycoproteins and determine their biosynthetic pathway.

## 4. Nucleotide Sugar Biosynthesis in Diatoms

**Figure 7:**
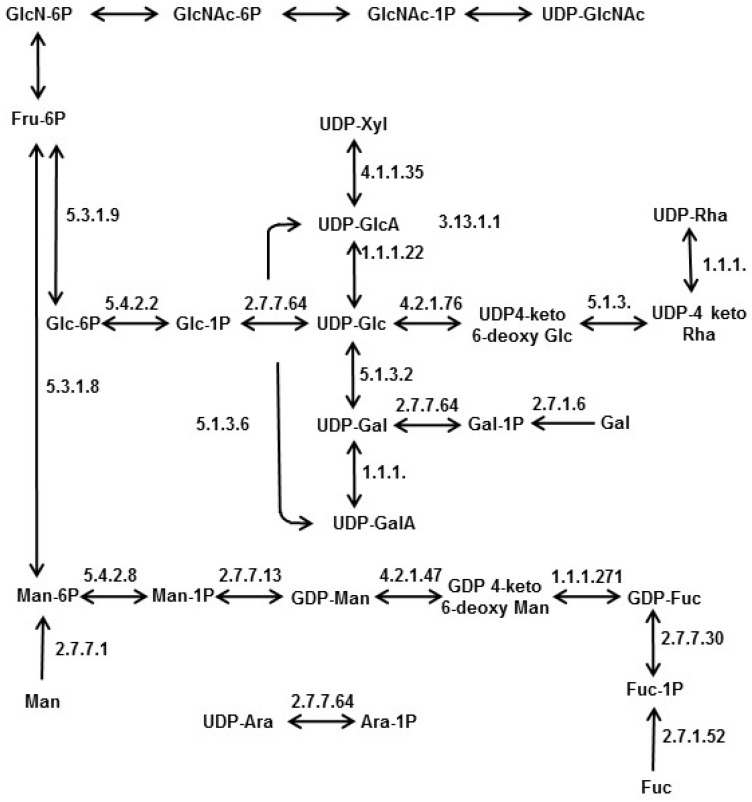
Predicted nucleotide sugar metabolism in diatoms based on bioinformatics analyses of the genomes from *Phaeodactylum tricornutum* [129], *Thalassiosira pseudonana* [130], *Fragilariopsis cylindrus* [131] and *Aureococcus anophagefferens* [132].

Monosaccharides represent the building blocks of glycans and polysaccharides (see Section 1). They are usually synthesized and converted into nucleotide sugars through a cytosolic interconversion metabolism (KEGG map 00520; [143]. In turn, nucleotide sugars—which are universal sugar donors—are involved in the formation of polysaccharides, glycoproteins, proteoglycans, and glycolipids. This metabolism is highly conserved in prokaryotes and eukaryotes and involves a set of phosphorylases, epimerases and reductases, as well as fructose-6-phosphate amino transferases enabling the synthesis of aminosugars. Nucleotide sugars may also result from the salvage pathway that involves the hydrolysis of glycans to free sugars, their phosphorylation and finally their nucleotidylation. Searches in diatom genomes (*P. tricornutum* [129], *T. pseudonana* [130], *Fragilariopsis cylindrus* [131], and *Aureococcus anophagefferens* [132] for genes encoding cytosolic enzymes of both the interconversion and salvage pathways have led to the identification of putative candidates for the synthesis of UDP-sugars such as UDP-galactose (UDP-Gal), UDP-galacturonic acid (UDP-GalA), UDP-glucuronic acid (UDP-GlcA), UDP-xylose (UDP-Xyl) and UDP-rhamnose (UDP-Rha) that are directly derived from UDP-Glc (Figure 7). Moreover, gene predictions also include enzymes required for the synthesis of the guanine diphosphate (GDP)-sugars originating from Man-6P (Figure 7). Aminosugar such as GlcNAc biosynthesis likely occurs by amination of the C2 on fructose-6P (Fru-6P) as reported in other organisms. Other gene predictions based on diatom genomes include genes encoding several sugar phosphorylases of the salvage pathway (Figure 7). However, neither UDP-galacturonate decarboxylases, nor UDP-arabinose 4-epimerases required for l-arabinose biosynthesis are predicted in these genomes. Other than arabinose, predicted nucleotide sugar metabolism is generally in agreement with the sugar compositions of polysaccharides and glycans isolated from diatoms.

## 5. Conclusions and Perspectives

The abundant literature cited in this review demonstrates that monosaccharide composition is highly variable in diatom EPSs. The variation in physiological conditions greatly influences their composition, suggesting that diatoms modulate their polysaccharide biosynthesis machinery to adapt to environmental conditions. EPSs have been shown to be heteropolysaccharides. Branched and sulfated glucuronomannans are thought to be ubiquitous and thus representative of diatom cell walls. β-chitin fibers have also been found in some diatom species. Chrysolaminarin is a common β(1,6) ramified β(1,3) polyglucan for food storage in diatoms. Although numerous efforts have been made to determine the structures of diatom polysaccharides, there is currently a lack of information regarding their biosynthesis pathways, as well as their cell localization and organization.

With regard to protein *N*-glycosylation, gene prediction analysis suggests that diatoms are equipped with most of the eukaryotic genes encoding ER-resident essential players, such as sugar transferases and chaperones. Some candidate genes involved in subsequent Golgi events of *N*-glycosylation have also been found in *P. tricornutum*, justifying further investigations and characterization of diatom *N*-glycosylation pathways. When looking at potential nucleotide sugar synthesis in diatom genomes, key enzyme genes were predicted for almost all monosaccharides, with the exception of those for arabinose synthesis, although this sugar was detected in some cell wall polysaccharides. In the coming years, the increasing demand for marine polysaccharides and for recombinant therapeutic glycoproteins produced in microalgae will lead the scientific community to carry out more research to better understand diatom polysaccharide and glycan structures, as well as their respective biosynthesis pathways and cell localizations.

## References

[1] Norton T.A., Melkonian M., Andersen R.A. (1996). Algal biodiversity. Phycologia.

[2] Falkowski P.G., Barber R.T., Smetacek V. (1998). Biogeochemical controls and feedbacks on ocean primary production. Science.

[3] Field C.B., Behrenfeld M.J., Randerson J.T., Falkowski P. (1998). Primary production of the biosphere: Integrating terrestrial and oceanic components. Science.

[4] Goldman J.C. (1993). Potential role of large oceanic diatoms in new primary production. Deep Sea Res. Part Oceanogr. Res. Pap..

[5] Caldwell G.S. (2009). The influence of bioactive oxylipins from marine diatoms on invertebrate reproduction and development. Mar. Drugs.

[6] Tréguer P., Nelson D.M., van Bennekom A.J., de Master D.J., Leynaert A., Quéguiner B. (1995). The silica balance in the world ocean: A reestimate. Science.

[7] Nelson D.M., Tréguer P., Brzezinski M.A., Leynaert A., Quéguiner B. (1995). Production and dissolution of biogenic silica in the ocean: Revised global estimates, comparison with regional data and relationship to biogenic sedimentation. Glob. Biogeochem. Cycles.

[8] Hutchins D.A., Bruland K.W. (1998). Iron-limited diatom growth and Si: N uptake ratios in a coastal upwelling regime. Nature.

[9] Kemp A.E.S., Pearce R.B., Grigorov I., Rance J., Lange C.B., Quilty P., Salter I. (2006). Production of giant marine diatoms and their export at oceanic frontal zones: Implications for Si and C flux from stratified oceans: Giant marine diatoms and their export. Glob. Biogeochem. Cycles.

[10] Allen A.E., LaRoche J., Maheswari U., Lommer M., Schauer N., Lopez P.J., Finazzi G., Fernie A.R., Bowler C. (2008). Whole-cell response of the pennate diatom *Phaeodactylum tricornutum* to iron starvation. Proc. Natl. Acad. Sci. USA.

[11] Sutak R., Botebol H., Blaiseau P.-L., Leger T., Bouget F.-Y., Camadro J.-M., Lesuisse E. (2012). A Comparative Study of Iron Uptake Mechanisms in Marine Microalgae: Iron Binding at the Cell Surface Is a Critical Step. Plant Physiol..

[12] Raven J.A. (2013). Iron acquisition and allocation in stramenopile algae. J. Exp. Bot..

[13] Collos Y. (1983). Transient situations in nitrate assimilation by marine diatoms. IV. Non-linear phenomena and the estimation of the maximum uptake rate. J. Plankton Res..

[14] Waser N.A.D., Harrison P.J., Nielsen B., Calvert S.E., Turpin D.H. (1998). Nitrogen isotope fractionation during the uptake and assimilation of nitrate, nitrite, ammonium, and urea by a marine diatom. Limnol. Oceanogr..

[15] Villareal T.A. (1989). Division cycles in the nitrogen-fixing *Rhizosolenia* (Bacillariophyceae)-*Richelia* (Nostocaceae) symbiosis. Br. Phycol. J..

[16] Weber T., Deutsch C. (2012). Oceanic nitrogen reservoir regulated by plankton diversity and ocean circulation. Nature.

[17] Craggs R.J., McAuley P.J., Smith V.J. (1997). Wastewater nutrient removal by marine microalgae grown on a corrugated raceway. Water Res..

[18] Lopez P.J., Desclés J., Allen A.E., Bowler C. (2005). Prospects in diatom research. Curr. Opin. Biotechnol..

[19] Lebeau T., Robert J.-M. (2003). Diatom cultivation and biotechnologically relevant products. Part II: Current and putative products. Appl. Microbiol. Biotechnol..

[20] Herlory O., Bonzom J.-M., Gilbin R., Frelon S., Fayolle S., Delmas F., Coste M. (2013). Use of diatom assemblages as biomonitor of the impact of treated uranium mining effluent discharge on a stream: Case study of the Ritord watershed (Center-West France). Ecotoxicology.

[21] Bræk G.S., Jensen A., Mohus Å. (1976). Heavy metal tolerance of marine phytoplankton. III. Combined effects of copper and zinc ions on cultures of four common species. J. Exp. Mar. Biol. Ecol..

[22] Morelli E., Pratesi E. (1997). Production of phytochelatins in the marine diatom *Phaeodactylum tricornutum* in response to copper and cadmium exposure. Bull. Environ. Contam. Toxicol..

[23] Pistocchi R., Mormile M.A., Guerrini F., Isani G., Boni L. (2000). Increased production of extra- and intracellular metal-ligands in phytoplankton exposed to copper and cadmium. J. Appl. Phycol..

[24] Parkinson J., Gordon R. (1999). Beyond micromachining: The potential of diatoms. Trends Biotechnol..

[25] Drum R.W., Gordon R. (2003). Star Trek replicators and diatom nanotechnology. Trends Biotechnol..

[26] Bozarth A., Maier U.-G., Zauner S. (2009). Diatoms in biotechnology: Modern tools and applications. Appl. Microbiol. Biotechnol..

[27] Jamali A.A., Akbari F., Ghorakhlu M.M., de la Guardia M., Khosroushahi A.Y. (2012). Applications of diatoms as potential microalgae in nanobiotechnology. BioImpacts.

[28] Muller-Feuga A. (2000). The role of microalgae in aquaculture: Situation and trends. J. Appl. Phycol..

[29] Becker E.W. (2007). Micro-algae as a source of protein. Biotechnol. Adv..

[30] Li H.-Y., Lu Y., Zheng J.-W., Yang W.-D., Liu J.-S. (2014). Biochemical and genetic engineering of diatoms for polyunsaturated fatty acid biosynthesis. Mar. Drugs.

[31] Kroth P. (2007). Molecular biology and the biotechnological potential of diatoms. Transgenic Microalgae as Green Cell Factories.

[32] Peng J., Yuan J.-P., Wu C.-F., Wang J.-H. (2011). Fucoxanthin, a marine carotenoid present in brown seaweeds and diatoms: Metabolism and bioactivities relevant to human health. Mar. Drugs.

[33] Kim S., Jung Y.-J., Kwon O.-N., Cha K., Um B.-H., Chung D., Pan C.-H. (2012). A potential commercial source of fucoxanthin extracted from the microalga *Phaeodactylum tricornutum*. Appl. Biochem. Biotechnol..

[34] Moreau D., Tomasoni C., Jacquot C., Kaas R., le Guedes R., Cadoret J.-P., Muller-Feuga A., Kontiza I., Vagias C., Roussis V. (2006). Cultivated microalgae and the carotenoid fucoxanthin from Odontella aurita as potent anti-proliferative agents in bronchopulmonary and epithelial cell lines. Environ. Toxicol. Pharmacol..

[35] Dalmo R., Martinsen B., Horsberg T., Ramstad A., Syvertsen C., Seljelid R., Ingebrigtsen K. (1998). Prophylactic effect of β(1,3)-d-glucan (laminaran) against experimental *Aeromonas salmonicida* and *Vibrio salmonicida infections*. J. Fish Dis..

[36] Sakai M. (1999). Current research status of fish immunostimulants. Aquaculture.

[37] Morales-Lange B., Bethke J., Schmitt P., Mercado L. (2014). Phenotypical parameters as a tool to evaluate the immunostimulatory effects of laminarin in *Oncorhynchus mykiss*. Aquac. Res..

[38] Skjermo J., Størseth T.R., Hansen K., Handå A., Øie G. (2006). Evaluation of β-(1 → 3,1 → 6)-glucans and High-M alginate used as immunostimulatory dietary supplement during first feeding and weaning of Atlantic cod (*Gadus morhua* L.). Aquaculture.

[39] Kusaikin M., Ermakova S., Shevchenko N., Isakov V., Gorshkov A., Vereshchagin A., Grachev M., Zvyagintseva T. (2010). Structural characteristics and antitumor activity of a new chrysolaminaran from the diatom alga *Synedra acus*. Chem. Nat. Compd..

[40] Raposo M., de Morais R., Bernardo de Morais A. (2013). Bioactivity and applications of sulphated polysaccharides from marine microalgae. Mar. Drugs.

[41] Abida H., Ruchaud S., Rios L., Humeau A., Probert I., de Vargas C., Bach S., Bowler C. (2013). Bioprospecting marine plankton. Mar. Drugs.

[42] Hempel F., Lau J., Klingl A., Maier U.G. (2011). Algae as protein factories: Expression of a human antibody and the respective antigen in the diatom *Phaeodactylum tricornutum*. PLoS ONE.

[43] Hempel F., Maier U.G. (2012). An engineered diatom acting like a plasma cell secreting human IgG antibodies with high efficiency. Microb. Cell Factories.

[44] Lingg N., Zhang P., Song Z., Bardor M. (2012). The sweet tooth of biopharmaceuticals: Importance of recombinant protein glycosylation analysis. Biotechnol. J..

[45] Van Beers M.M.C., Bardor M. (2012). Minimizing immunogenicity of biopharmaceuticals by controlling critical quality attributes of proteins. Biotechnol. J..

[46] Nguema-Ona E., Vicré-Gibouin M., Gotté M., Plancot B., Lerouge P., Bardor M., Driouich A. (2014). Cell wall *O*-glycoproteins and *N*-glycoproteins: Aspects of biosynthesis and function. Front. Plant Sci..

[47] Bar-Peled M., O’Neill M.A. (2011). Plant Nucleotide Sugar Formation, Interconversion, and Salvage by Sugar Recycling. Annu. Rev. Plant Biol..

[48] Hoagland K.D., Rosowski J.R., Gretz M.R., Roemer S.C. (1993). Diatom extracellular polymeric substances: Function, fine structure, chemistry, and physiology. J. Phycol..

[49] Underwood G.J.C., Paterson D.M. (2003). The importance of extracellular carbohydrate production by marine epipelic diatoms. Adv. Bot. Res..

[50] Chiovitti A., Higgins M.J., Harper R.E., Wetherbee R., Bacic A. (2003). The complex polysaccharides of the raphid diatom *Pinnularia viridis* (Bacillariophyceae). J. Phycol..

[51] Chiovitti A., Molino P., Crawford S.A., Teng R., Spurck T., Wetherbee R. (2004). The glucans extracted with warm water from diatoms are mainly derived from intracellular chrysolaminaran and not extracellular polysaccharides. Eur. J. Phycol..

[52] Chiovitti A., Harper R.E., Willis A., Bacic A., Mulvaney P., Wetherbee R. (2005). Variations in the substituted 3-linked mannans closely associated with the silicified walls of diatoms: Substituted mannans of diatoms. J. Phycol..

[53] Tesson B., Hildebrand M. (2013). Characterization and localization of insoluble organic matrices associated with diatom cell walls: Insight into their roles during cell wall formation. PLoS ONE.

[54] Nakajima T., Volcani B. (1969). 3,4-Dihydroxyproline: A new amino acid in diatom cell walls. Science.

[55] Hecky R.E., Mopper K., Kilham P., Degens E.T. (1973). The amino acid and sugar composition of diatom cell-walls. Mar. Biol..

[56] Swift D.M., Wheeler A. (1992). Evidence of an organic matrix from diatom biosilica. J. Phycol..

[57] Kröger N. (1999). Polycationic peptides from diatom biosilica that direct silica nanosphere formation. Science.

[58] Volcani B.E., Simpson T., Volcani B. (1981). Cell wall formation in diatoms: Morphogenesis and biochemistry. Silicon and Siliceous Structures in Biological Systems.

[59] Kröger N., Poulsen N. (2008). Diatoms from cell wall biogenesis to nanotechnology. Annu. Rev. Genet..

[60] Kates M., Volcani B. (1968). Studies on the biochemistry and fine structure of silica shell formation in diatoms. Lipid components of the cell walls. Z. Pflanzenphysiol..

[61] Tesson B., Genet M.J., Fernandez V., Degand S., Rouxhet P.G., Martin-Jézéquel V. (2009). Surface chemical composition of diatoms. ChemBioChem.

[62] Haug A., Myklestad S. (1976). Polysaccharides of marine diatoms with special reference to *Chaetoceros* species. Mar. Biol..

[63] Cowie G.L., Hedges J.I. (1996). Digestion and alteration of the biochemical constituents of a diatom (*Thalassiosira weisflogii*) ingested by an herbivorous zooplankton (*Calanus pacificus*). Limnol. Oceanogr..

[64] Coombs J., Volcani B. (1968). Studies on the biochemistry and fine structure of silica shell formation in diatoms. Planta.

[65] Ford C.W., Percival E. (1965). Carbohydrates of *Phaeodactylum tricornutum*. Part II. A sulphated glucuronomannan. J. Chem. Soc..

[66] Abdullahi A.S., Underwood G.J.C., Gretz M.R. (2006). Extracellular matrix assembly in diatoms (Bacillariophyceae). V. Environmental effects on polysaccharide synthesis in the model diatom, *Phaeodactylum tricornutum*. J. Phycol..

[67] Willis A., Chiovitti A., Dugdale T.M., Wetherbee R. (2013). Characterization of the extracellular matrix of *Phaeodactylum tricornutum* (Bacillariophyceae): Structure, composition, and adhesive characteristics. J. Phycol..

[68] McConville M.J., Wetherbee R., Bacic A. (1999). Subcellular location and composition of the wall and secreted extracellular sulphated polysaccharides/proteoglycans of the diatom *Stauroneis amphioxys* Gregory. Protoplasma.

[69] Allan G.G., Lewin J., Johnson P.G. (1972). Marine polymers. IV Diatom polysaccharides. Bot. Mar..

[70] Dweltz N., Colvin J.R., McInnes A. (1968). Studies on chitan (β-(1 → 4)-linked 2-acetamido-2-deoxy-d-glucan) fibers of the diatom *Thalassiosira fluviatilis*, Hustedt. III. The structure of chitan from X-ray diffraction and electron microscope observations. Can. J. Chem..

[71] Blackwell J., Parker K.D., Rudall K.M. (1967). Letter to the Editor: Chitin fibres of the diatoms *Thalassiosira fluviatilis* and *Cyclotella cryptica*. J. Mol. Biol..

[72] Lindsay G.J., Gooday G.W. (1985). Action of chitinase on spines of the diatom *Thalassiosira fluviatilis*. Carbohydr. Polym..

[73] Nishiyama Y., Noishiki Y., Wada M. (2011). X-ray Structure of Anhydrous β-Chitin at 1 Å Resolution. Macromolecules.

[74] Sawada D., Nishiyama Y., Langan P., Forsyth V.T., Kimura S., Wada M. (2012). Water in crystalline fibers of dihydrate β-chitin results in unexpected absence of intramolecular hydrogen bonding. PLoS ONE.

[75] Blackwell J., Parker K.D., Rudall K.M. (1965). Chitin in pogonophore tubes. J. Mar. Biol. Assoc. UK.

[76] Gaill F., Persson J., Sugiyama J., Vuong R., Chanzy H. (1992). The chitin system in the tubes of deep sea hydrothermal vent worms. J. Struct. Biol..

[77] Ogawa Y., Kimura S., Wada M. (2011). Electron diffraction and high-resolution imaging on highly-crystalline β-chitin microfibril. J. Struct. Biol..

[78] Herth W. (1978). A special chitin-fibril-synthesizing apparatus in the centric diatom *Cyclotella*. Naturwissenschaften.

[79] Herth W. (1979). The site of β-chitin fibril formation in centric diatoms. II. The chitin-forming cytoplasmic structures. J. Ultrastruct. Res..

[80] Herth W., Barthlott W. (1979). The site of β-chitin fibril formation in centric diatoms. I. Pores and fibril formation. J. Ultrastruct. Res..

[81] Shillito B., Koster A.J., Walz J., Baumeister W. (1996). Electron tomographic reconstruction of plastic-embedded organelles involved in the chitin secretion process. Biol. Cell.

[82] Ravaux J., Shillito B., Gaill F., Gay L., Voss-Foucart M.-F., Childress J. (1998). Tube synthesis and growth processes in the hydrothermal vent tube-worm *Riftia pachyptila*. Cah. Biol. Mar..

[83] Sugiyama J., Boisset C., Hashimoto M., Watanabe T. (1999). Molecular directionality of β-chitin biosynthesis. J. Mol. Biol..

[84] Imai T., Watanabe T., Yui T., Sugiyama J. (2003). The directionality of chitin biosynthesis: A revisit. Biochem. J..

[85] Durkin C.A., Mock T., Armbrust E.V. (2009). Chitin in diatoms and its association with the cell wall. Eukaryot. Cell.

[86] Tesson B., Masse S., Laurent G., Maquet J., Livage J., Martin-Jézéquel V., Coradin T. (2008). Contribution of multi-nuclear solid state NMR to the characterization of the *Thalassiosira pseudonana* diatom cell wall. Anal. Bioanal. Chem..

[87] Morin L.G., Smucker R.A., Herth W. (1986). Effects of two chitin synthesis inhibitors on *Thalassiosira fluviatilis* and *Cyclotella cryptica*. FEMS Microbiol. Lett..

[88] Quillet M., Combes R. (1955). Sur la nature chimique de la leucosine, polysaccharide de réserve caractéristique des Chrysophycées, extraite d’*Hydrudus foetidus*. C. R. Hebd. Séances Acad. Sci..

[89] Waterkeyn L., Bienfait A. (1987). Localization and function of β(1,3)-glucans (callose and chrysolaminarin) in *Pinnularia* genus (Diatoms). Cellule (Belg.).

[90] Myklestad S. (1974). Production of carbohydrates by marine planktonic diatoms. I. Comparison of nine different species in culture. J. Exp. Mar. Biol. Ecol..

[91] Beattie A., Hirst E.L., Percival E. (1961). Studies on the metabolism of the Chrysophyceae. Biochem. J..

[92] Ford C.W., Percival E. (1965). The carbohydrates of *Phaeodactylum tricornutum*. Part I. Preliminary examination of the organism, and characterisation of low molecular weight material and of a glucan. J. Chem. Soc..

[93] Alekseeva S.A., Shevchenko N.M., Kusaykin M.I., Ponomorenko L.P., Isakov V.V., Zvyagintseva T.N., Likhoshvai E.V. (2005). Polysaccharides of diatoms occurring in Lake Baikal. Appl. Biochem. Microbiol..

[94] Størseth T.R., Hansen K., Reitan K.I., Skjermo J. (2005). Structural characterization of β-d-(1 → 3)-glucans from different growth phases of the marine diatoms *Chaetoceros mülleri* and *Thalassiosira weissflogii*. Carbohydr. Res..

[95] Kim Y.-T., Kim E.-H., Cheong C., Williams D.L., Kim C.-W., Lim S.-T. (2000). Structural characterization of β-d-(1 → 3, 1 → 6)-linked glucans using NMR spectroscopy. Carbohydr. Res..

[96] Paulsen B.S., Myklestad S. (1978). Structural studies of the reserve glucan produced by the marine diatom *Skeletonema costatum* (Grev.) Cleve. Carbohydr. Res..

[97] McConville M.J., Bacic A., Clarke A.E. (1986). Structural studies of chrysolaminaran from the ice diatom *Stauroneis amphioxys* (Gregory). Carbohydr. Res..

[98] Wustman B.A., Gretz M.R., Hoagland K.D. (1997). Extracellular matrix assembly in diatoms (Bacillariophyceae). I. A model of adhesives based on chemical characterization and localization of polysaccharides from the marine diatom *Achnanthes longipes* and other diatoms. Plant Physiol..

[99] Chiovitti A., Bacic A., Burke J., Wetherbee R. (2003). Heterogeneous xylose-rich glycans are associated with extracellular glycoproteins from the biofouling diatom *Craspedostauros australis* (Bacillariophyceae). Eur. J. Phycol..

[100] Størseth T.R., Kirkvold S., Skjermo J., Reitan K.I. (2006). A branched β-d-(1 → 3, 1 → 6)-glucan from the marine diatom *Chaetoceros debilis* (Bacillariophyceae) characterized by NMR. Carbohydr. Res..

[101] Lee J.Y., Kim Y.-J., Kim H.J., Kim Y.-S., Park W. (2012). Immunostimulatory effect of laminarin on RAW 264.7 mouse macrophages. Molecules.

[102] De Brouwer J., Wolfstein K., Stal L.J. (2002). Physical characterization and diel dynamics of different fractions of extracellular polysaccharides in an axenic culture of a benthic diatom. Eur. J. Phycol..

[103] Underwood G.J.C., Boulcott M., Raines C.A., Waldron K. (2004). Environmental effects on exopolymer production by marine benthic diatoms: Dynamics, changes in composition, and pathways of production: Exopolymer production by diatoms. J. Phycol..

[104] Bellinger B., Abdullahi A., Gretz M., Underwood G. (2005). Biofilm polymers: Relationship between carbohydrate biopolymers from estuarine mudflats and unialgal cultures of benthic diatoms. Aquat. Microb. Ecol..

[105] Hanlon A.R.M., Bellinger B., Haynes K., Xiao G., Hofmann T.A., Gretz M.R., Ball A.S., Osborn A.M., Underwood G.J.C. (2006). Dynamics of extracellular polymeric substance (EPS) production and loss in an estuarine, diatom-dominated, microalgal biofilm over a tidal emersio-immersion period. Limnol. Oceanogr..

[106] Penna A., Berluti S., Penna N., Magnani M. (1999). Influence of nutrient ratios on the *in vitro* extracellular polysaccharide production by marine diatoms from the Adriatic Sea. J. Plankton Res..

[107] Staats N., de Winder B., Stal L., Mur L. (1999). Isolation and characterization of extracellular polysaccharides from the epipelic diatoms *Cylindrotheca closterium* and *Navicula salinarum*. Eur. J. Phycol..

[108] Magaletti E., Urbani R., Sist P., Ferrari C.R., Cicero A.M. (2004). Abundance and chemical characterization of extracellular carbohydrates released by the marine diatom *Cylindrotheca fusiformis* under *N*-and *P*-limitation. Eur. J. Phycol..

[109] Urbani R., Magaletti E., Sist P., Cicero A.M. (2005). Extracellular carbohydrates released by the marine diatoms *Cylindrotheca closterium*, *Thalassiosira pseudonana* and *Skeletonema costatum*: Effect of *P*-depletion and growth status. Sci. Total Environ..

[110] Ai X.-X., Liang J.-R., Gao Y.-H., Lo S.C.-L., Lee F.W.-F., Chen C.-P., Luo C.-S., Du C. (2015). MALDI-TOF MS analysis of the extracellular polysaccharides released by the diatom *Thalassiosira pseudonana* under various nutrient conditions. J. Appl. Phycol..

[111] Wustman B.A., Lind J., Wetherbee R., Gretz M.R. (1998). Extracellular matrix assembly in diatoms (Bacillariophyceae) III. Organization of fucoglucuronogalactans within the adhesive stalks of Achnanthes longipes. Plant Physiol..

[112] Takahashi E., Ledauphin J., Goux D., Orvain F. (2009). Optimising extraction of extracellular polymeric substances (EPS) from benthic diatoms: Comparison of the efficiency of six EPS extraction methods. Mar. Freshw. Res..

[113] Zhang S., Xu C., Santschi P.H. (2008). Chemical composition and 234Th (IV) binding of extracellular polymeric substances (EPS) produced by the marine diatom *Amphora* sp.. Mar. Chem..

[114] Leandro S.M., Gil M.C., Delgadillo I. (2003). Partial characterisation of exopolysaccharides exudated by planktonic diatoms maintained in batch cultures. Acta Oecol..

[115] Khandeparker R.D., Bhosle N.B. (2001). Extracellular polymeric substances of the marine fouling diatom *Amphora rostrata Wm.Sm*. Biofouling.

[116] Myklestad S., Haug A., Larsen B. (1972). Production of carbohydrates by the marine diatom *Chaetoceros affinis var. willei* (Gran) Hustedt. II. Preliminary investigation of the extracellular polysaccharide. J. Exp. Mar. Biol. Ecol..

[117] Smestad B., Haug A., Myklestad S. (1974). Production of carbohydrate by the marine diatom *Chaetoceros affinis var. willei* (Gran) Hustedt. III. Structural studies of the extracellular polysaccharide. Acta Chem. Scand. B.

[118] Smestad B., Haug A., Myklestad S. (1975). Structural studies of the extracellular polysaccharide produced by the diatom *Chaeotoceros curvisetus* Cleve. Acta Chem. Scand. B.

[119] Percival E., Rahman M.A., Weigel H. (1980). Chemistry of the polysaccharides of the diatom *Coscinodiscus nobilis*. Phytochemistry.

[120] Bhosle N., Sawant S., Garg A., Wagh A. (1995). Isolation and partial chemical analysis of exopolysaccharides from the marine fouling diatom *Navicula subinflata*. Bot. Mar..

[121] Giroldo D., Vieira A.A.H., Paulsen B.S. (2003). Relative increase of deoxy sugars during microbial degradation of an extracellular polysaccharide released by a tropical freshwater *Thalassiosira* sp. (Bacillariophyceae). J. Phycol..

[122] Varki A., Cummings R.D., Esko J.D., Freeze H.H., Stanley P., Marth J.D., Bertozzi C.R., Hart G.W., Etzler M.E. (2009). Symbol nomenclature for glycan representation. Proteomics.

[123] Burda P., Aebi M. (1999). The dolichol pathway of *N*-linked glycosylation. Biochim. Biophys. Acta (BBA) Gen. Subj..

[124] Gil G.-C., Velander W.H., van Cott K.E. (2009). *N*-glycosylation microheterogeneity and site occupancy of an Asn-X-Cys sequon in plasma-derived and recombinant protein C. Proteomics.

[125] Zielinska D.F., Gnad F., Wiśniewski J.R., Mann M. (2010). Precision mapping of an *in vivo*
*N*-glycoproteome reveals rigid topological and sequence constraints. Cell.

[126] Matsui T., Takita E., Sato T., Kinjo S., Aizawa M., Sugiura Y., Hamabata T., Sawada K., Kato K. (2011). *N*-glycosylation at noncanonical Asn-X-Cys sequences in plant cells. Glycobiology.

[127] Helenius A., Aebi M. (2004). Roles of *N*-linked glycans in the endoplasmic reticulum. Annu. Rev. Biochem..

[128] Weerapana E., Imperiali B. (2006). Asparagine-linked protein glycosylation: From eukaryotic to prokaryotic systems. Glycobiology.

[129] Bowler C., Allen A.E., Badger J.H., Grimwood J., Jabbari K., Kuo A., Maheswari U., Martens C., Maumus F., Otillar R.P. (2008). The *Phaeodactylum* genome reveals the evolutionary history of diatom genomes. Nature.

[130] Armbrust E.V., Berges J.A., Bowler C., Green B.R., Martinez D., Putnam N.H., Zhou S., Allen A.E., Apt K.E., Bechner M. (2004). The genome of the diatom *Thalassiosira pseudonana*: Ecology, evolution, and metabolism. Science.

[131] JGI Genome Portal, *Fragilariopsis cylindrus* Home. http://genome.jgi-psf.org/Fracy1/Fracy1.home.html.

[132] Gobler C.J., Berry D.L., Dyhrman S.T., Wilhelm S.W., Salamov A., Lobanov A.V., Zhang Y., Collier J.L., Wurch L.L., Kustka A.B. (2011). Niche of harmful alga *Aureococcus anophagefferens* revealed through ecogenomics. Proc. Natl. Acad. Sci. USA.

[133] Baiet B., Burel C., Saint-Jean B., Louvet R., Menu-Bouaouiche L., Kiefer-Meyer M.-C., Mathieu-Rivet E., Lefebvre T., Castel H., Carlier A. (2011). *N*-Glycans of *Phaeodactylum tricornutum* diatom and functional characterization of Its *N*-acetylglucosaminyl transferase I enzyme. J. Biol. Chem..

[134] Mathieu-Rivet E., Kiefer-Meyer M.-C., Vanier G., Ovide C., Burel C., Lerouge P., Bardor M. (2014). Protein *N*-glycosylation in eukaryotic microalgae and its impact on the production of nuclear expressed biopharmaceuticals. Front. Plant Sci..

[135] Levy-Ontman O., Fisher M., Shotland Y., Weinstein Y., Tekoah Y., Arad S. (2014). Genes involved in the endoplasmic reticulum *N*-glycosylation pathway of the red microalga *Porphyridium* sp.: A bioinformatic study. Int. J. Mol. Sci..

[136] Varki A. (2011). Evolutionary forces shaping the Golgi glycosylation machinery: Why cell surface glycans are universal to living cells. Cold Spring Harb. Perspect. Biol..

[137] Varki A. (1993). Biological roles of oligosaccharides: All of the theories are correct. Glycobiology.

[138] Gagneux P., Varki A. (1999). Evolutionary considerations in relating oligosaccharide diversity to biological function. Glycobiology.

[139] Vitale A., Chrispeels M.J. (1984). Transient *N*-acetylglucosamine in the biosynthesis of phytohemagglutinin: Attachment in the Golgi apparatus and removal in protein bodies. J. Cell Biol..

[140] Altmann F., Schwihla H., Staudacher E., Glössl J., März L. (1995). Insect cells contain an unusual, membrane-bound-*N*-acetylglucosaminidase probably involved in the processing of protein *N*-glycans. J. Biol. Chem..

[141] Kröger N., Bergsdorf C., Sumper M. (1994). A new calcium binding glycoprotein family constitutes a major diatom cell wall component. EMBO J..

[142] Lind J.L., Heimann K., Miller E.A., van Vliet C., Hoogenraad N.J., Wetherbee R. (1997). Substratum adhesion and gliding in a diatom are mediated by extracellular proteoglycans. Planta.

[143] KEGG PATHWAY Database. http://www.genome.jp/kegg/pathway.html.

